# Genetics of catatonia: a systematic review of case reports and a gene pathway analysis

**DOI:** 10.1192/j.eurpsy.2025.2458

**Published:** 2025-05-22

**Authors:** Mylene Moyal, Anton Iftimovici, Wafa Ghoul, Marion Plaze, Boris Chaumette

**Affiliations:** 1 GHU-Paris Psychiatrie et Neurosciences, Hôpital Sainte Anne, Paris, France; 2 Université Paris Cité, Institute of Psychiatry and Neuroscience of Paris (IPNP), INSERM U1266, Paris, France; 3 Human Genetics and Cognitive Functions, Institut Pasteur, CNRS UMR3571, Université Paris Cité, Paris, France; 4 Department of Psychiatry, McGill University, Montreal, Canada

**Keywords:** catatonic syndrome, excitation/inhibition imbalance, GABAergic interneurons, genetic, neurodevelopmental, variants

## Abstract

**Background:**

Neurodevelopmental conditions are crucial risk factors for catatonia in pediatric and adult populations. Recent case reports and studies have identified an increasing number of genetic abnormalities likely contributing to catatonia. Catatonia associated with genetic abnormalities is challenging in terms of identification, chronicity, and resistance to treatment. In addition, understanding these genetic abnormalities through identifying rare single nucleotide and copy number variants may offer valuable insights into the underlying pathophysiology.

**Methods:**

We conducted a systematic review of all genetic abnormalities reported with catatonia and performed a gene-set enrichment analysis. Our systematic literature search for relevant articles published through July 15, 2024, using combinations of “catatonia,” “catatonic syndrome,” “genetic,” and “genes” in PubMed, yielded 317 articles. Of these, 94 were included, covering 374 cases of catatonia and 78 distinct genetic abnormalities.

**Results:**

This review discusses the clinical presentation of catatonia for each genetic disorder, the treatment strategies, and the putative underlying mechanisms.

**Conclusions:**

The review highlights that catatonia underpinned by genetic abnormalities presents specific clinical and treatment-response features. Therefore, we propose genetic testing guidelines for catatonia and advocate for systematically investigating catatonia in several genetic diseases. Regarding the pathophysiology of catatonia, the gene ontology of biological processes reveals significant enrichment of variants in synaptic and post-synaptic regulatory genes, particularly within GABAergic neurons, reinforcing the implication of the excitatory/inhibitory imbalance. Finally, genetic variants are enriched in microglial cells, highlighting the role of brain inflammation in triggering catatonia. This comprehensive insight could pave the way for more effective management strategies for this condition.

## Introduction

Catatonia is a life-threatening psychomotor syndrome observed in psychiatric disorders, such as psychotic or mood disorders, alongside other medical conditions [1]. It is common in adult psychiatric wards, with a prevalence ranging from 9 to 30% [1]. In children and young adults, prevalence varies from 0.6 to 17% [2]. Moreover, its prevalence among neurodevelopmental disorders is increasingly recognized [3, 4], and imaging studies point to anomalies in brain development (i.e. deviation of sulcation and gyrification indexes) [5, 6] as a critical risk factor for its emergence in pediatric and adult populations [2]. By proxy, genetic anomalies reported in neurodevelopmental conditions have also been identified in catatonia with neurodevelopmental features [7] and in pediatric populations [8]. In addition, these neurodevelopmentally associated-catatonia tend to exhibit heightened complexity in identification, chronicity, and resistance to treatment [2, 9, 10]. Indeed, besides the DSM-5 criteria for catatonia (i.e. catalepsy, stupor, waxy flexibility, agitation, mutism, negativism, posturing, mannerisms, stereotypies, grimacing and echophenomena [11]), the onset of incontinence, regression in acquisitions or worsening of pre-existing symptoms such as stereotypies and self-injurious behaviors are frequent features of neurodevelopmentally associated-catatonia [2]. Notably, the established diagnostic tool for catatonia, the Bush-Francis Catatonia Rating Scale (BFCRS) [12], lacks consideration of specific clinical signs often observed in these cases, such as functional regression [2]. Within this neurodevelopmental context, the catatonic episodes tend to be chronic, lasting more than 12 weeks [13] and show suboptimal response to first-line treatments, lorazepam and electroconvulsive therapy (ECT) [9, 14], due to poor drug tolerance but also because the physical condition limits access to ECT [15, 16]. Identifying and treating catatonic episodes linked to genetic anomalies is, therefore, a significant healthcare problem that needs to be addressed.

In addition, genetic abnormalities offer valuable insights into the underlying pathophysiology [3, 17]. Genome-wide association studies (GWAS) conducted on schizophrenia, bipolar disorder, and autism spectrum disorder have pinpointed various genetic polymorphisms linked to these conditions [18]. Some of these genes significantly influence neurotransmitter signaling, brain development, and synaptic function. An initial GWAS study involving 119 catatonia patients failed to unveil any specific single nucleotide polymorphism (SNPs) tied to catatonia [19], mainly due to the limited sample size. Much can be gained by investigating the rare variants that have been extensively reported in neurodevelopmental condition [20]. A 2018 literature review [21] and a recent genetic study [7] highlighted a wide range of genetic abnormalities associated with catatonia with several variants implicated in the GABA/glutamate pathway. Excitation/inhibition (E/I) imbalance is a strong hypothesis in the pathophysiology of catatonia that is supported by the efficacy of GABA_A_ agonists (such as lorazepam) and neuroimaging studies [22, 23]. Moreover, emerging evidence suggests an autoimmune facet to catatonia [24, 25], wherein genetic elements associated with immune system dysregulation and dysimmunity could contribute.

Hence, characterizing genetic abnormalities may provide valuable insights into catatonia’s underlying mechanisms and pave the way for more effective management strategies for these challenging, treatment-resistant catatonic episodes. In this systematic review, we aim to update the subject with a systematic review of all the genetic abnormalities reported as leading to catatonia in light of the increasing number of case reports over the last years and to perform a gene enrichment analysis to refine our understanding of the pathophysiology.

## Methods

**Figure 1.**
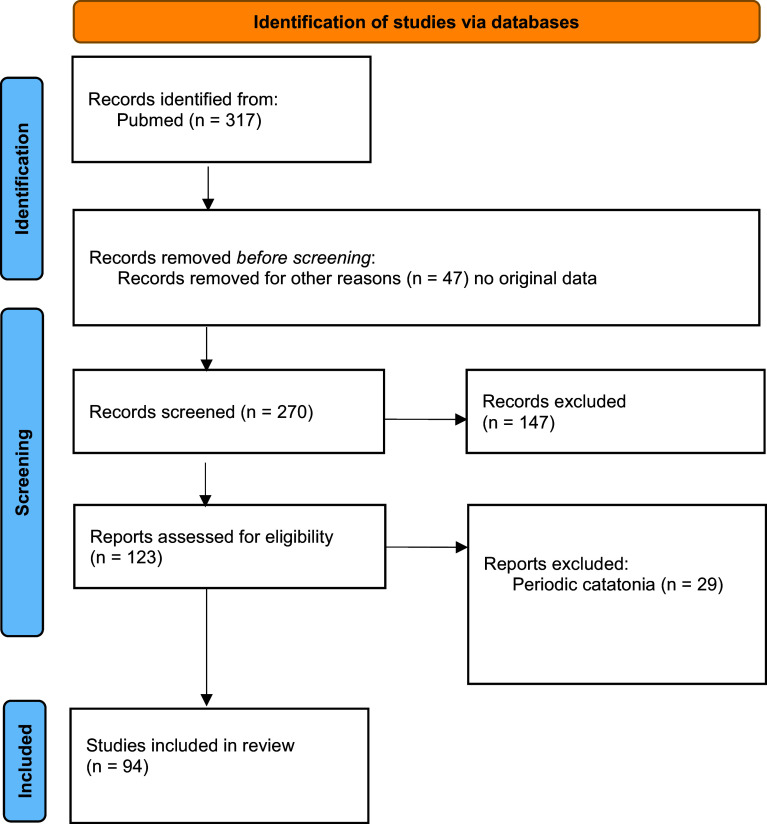
Flow chart.

A systematic literature search was conducted for articles, including case reports, describing subjects with catatonia and genetic abnormalities. We applied the Preferred Reporting Items for Systematic Reviews and Meta-Analysis (PRISMA) guideline [26] and declared the review in Prospero (ID: CRD42024526691). We used PubMed and followed up on the references cited in the papers that were thus identified. The following keywords were used: (catatonia or catatonic syndrome) and (genes or genetic or variants or mutations). All relevant articles published up to July 15, 2024, were included (Figure 1). The database search was done on 15 July 2024. Papers were included in the systematic review if (a) they were published in an English-language peer-reviewed journal; (b) the study enrolled patients with catatonia; (c) the study described one or more cases of patients with catatonia and genetic anomalies. We excluded reviews and case series that did not provide data on individual patients. Articles in line with Wernicke–Kleist–Leonhard catatonia (*N* = 29) were also excluded from the analysis as the definition does not fit the DSM-5 catatonia criteria [11]. Following PRISMA guidelines, two different reviewers (MM and WG) searched, selected, and included articles. In case of disagreement, the other authors had to make the decision.

For each report, we collected the following information: age, underlying pathology, episode characteristics, treatment used, and the catatonia diagnostic scale (Table 1). All data were extracted on 20 July 2024.

**Table 1.** Genetic abnormalities reported in catatonia

Genetic anomaly	Genes implicated	Catatonia case	Age	Scale	Treatment	References
21 Trisomy (Down syndrome)	Multiple	142 cases	adolescence	/	Lorazepam and ECT in some cases. tDCS in 2 cases. IVIg effective	[7, 8, 31, 33, 38, 110–122]
22q11.2 deletion (DiGeorge syndrome) and 1 duplication	Multiple	21 cases	/	/	Lorazepam and ECT in some cases. 2 were unsuccessfully treated with IVIg	[21, 38, 46, 114, 123]
22q13.3 deletion or mutation (Phelan Mc-Dermid syndrome)	*SHANK* 3	51 cases	adolescence	/	Lorazepam not tolerated Lithium in some cases ECT in some cases tDCS in 4 cases	[7, 8, 37, 38, 40, 41, 124–133]
15q11-q13 (Prader-Willi syndrome)	Multiple	9 cases	/	BFCRS	Lorazepam + ECT	[55, 56, 134, 135]
Huntington disease CAG repeat	*HTT*	6 cases mood disorder or psychosis	57; 20; 26; 62; 38; 16	BFCRS; PCRS	ECT effective	[8, 59, 136–138]
Kleefstra syndrome	*EHMT1*	6 cases	18, /	PCRS ; /	/	[8, 49]
Heterozygous pathogenic variant	*GABRB2*	1 case ASD ID and chronic catatonia	17	DSM5 + BFCRS	Lorazepam + ECT	[139]
Pathogenic frameshift variant	*HIVEP2*	2 cases including 1 MC	22	/	1 case: Lorazepam no effect amantadine badly tolerated Celecoxib + quetiapine effective Other case: MC treated with ECT	[140, 141]
Heterozygous full deletion	*VAMP2*	1 MC	33	/	Lorazepam not tolerated Treated with ECT	[142]
Baraitser Winter syndrome	*ACTB* and *ACTG1*	2 cases	18	/	/	[143]
Heterozygote pathogenic variant	*CACNA1A; CACNA1D*	1 case of dementia 1 case with adverse effect on ECT	9; 14	/	Lorazepam not sufficient. Treated with ECT	[144, 145]
15q duplication	Multiple	1 case with ASD	/	DSM5	ECT	[114]
3q13.13 intragenic deletion	/	1 case with ASD	/	DSM5	ECT	[114]
9p24.2 deletion	/	1 case with ASD	/	DSM5	ECT	[114]
X28q duplication	/	1 case with ASD	/	DSM5	ECT	[114]
Pathogenic variant	*SYNJ1*	1 case co-occurring with PMS with ID and recurrent catatonia	19	/	Alprazolam, Lithium, and Levodopa	[126]
Stop-gain mutation	*RCL1*	1 case with very early onset psychosis	14	DSM5	Clonazepam	[146]
Heterozygous variant	*mTOR*	1 case ID and ASD	19	BFCRS	Lorazepam not tolerated Treated with ECT	[147]
Pitt-Hopkins Syndrome	*TCF4*	1 case ID and ASD	23	/	ECT	[148]
De novo missense variant	*NLGN2*	1 case with ASD and chronic catatonia	21	/	Clonazepam + aripiprazole	[149]
Aicardi-Goutières syndrome homozygous	*RNASEH2B* *SAMHD1*	1 case with leucoencephalitis. 1 case with ID, psychosis and Degos like skin lesions	15; 19	DSM5 + BFCRS	treated with methylprednisolone then prednisone combined with mycophenolate mofetil and hydroxychloroquine then immunoadsorption (22 courses)	[101, 102]
Missense variant	*TBK1*	1 case of catatonia with post infection psychosis	/	/	/	[105]
Rett syndrome	*MECP2*	1 case with ID early-onset parkinsonism, and VSGP + 1 case male with epilepsy + 1 catatonia and regression + 20 catatonia like behavior	17; 17	/ DSM–5 and BFCRS; ABQ; PCRS	Lorazepam effective; tDCS; lorazepam effective	[2, 68–70]
S93L mutation	*MLC1*	1 case with bipolar disorder and catatonia	17	/	Olanzapine + Sertraline	[150]
Fragile X syndrome trinucleotide repeat expansion CGG >200 and 1 premutation career	*FMR1*	1 case with ADHD and ASD 1 case with neuro-lupus 1 premutation career with ADHD, ASD, BP, OCD, Tourette syndrome and recurrent chronic catatonia + 8 cases of catatonia like behavior	18; 21; 25; /	DSM–5 BFCRS ABQ	1 case: Lorazepam partial improvement 3 cases treated with ECT 8 cases: /	[62–65]
Cornelia de Langes syndrome	Multiple	10 cases of catatonia like behavior	/	ABQ	/	[63]
15q11 with SNORD 115 mRNA	*CRHR1, PBRM1, TAF1, DPM2, RALGPS1*	3 cases	9; /; /	/	Lorazepam effective for one case	[57]
Fatal familial insomnia (FFI)	*PRNP*	1 case with mood disorder, and insomnia	18	/	Worse with BZD and ECT	[8, 151]
4 missense mutations (P406L, R431H, Q19P, R185W)	*PRODH*	1 case with ASD, ID, schizophrenia and seizures	14	PCRS	/	[8]
Mucopolysaccharidosis de type III (Sanfilippo syndrome)	Multiple	1 case with ID, ASD and dementia	18	PCRS	/	[8]
16p13.1 duplication	*PDXDC1, NTAN1, RRN3, PKD1P6-NPIPP1, NPIPA5, MPV17L, BMERB1, MARF1, NDE1, MYH11, ABCC1, ABCC6*	1 case with ASD, schizophrenia and catatonia	16	PCRS	/	[8]
8p23.3 deletion	*DLGAP2, CNL8*	1 case with ASD	17	PCRS	/	[8]
2q22.1 deletion	*THSD7B*	1 case with ID, ASD and seizures	19	PCRS	/	[8]
13q33.1–34 deletion	*DAOA, EFNB2, ARGLU1, FAM155A, LIG4, ABHD13, TNFSF13B, MYO16, IRS2*	1 case with hemostatic disorder, ID, and seizures	14	PCRS	/	[8]
2q36 deletion	*DOCK10, NYAP2*	1 case with ID and ASD, seizures	15	PCRS	/	[8]
6q25.2–27 deletion	*PRKN*	1 case with anti-NMDA receptor encephalitis	17	PCRS	/	[8]
Xp22.33 duplication	*CRLF2, ASMTL, PERY8*	1 case with ID, ASD, and seizures	19	PCRS	/	[8]
21q21.1 duplication 6q14 duplication Xq25 deletion	No coding gene No coding gene No coding gene	1 case with ID, BP	14	PCRS	/	[8]
Ala202Val; Gln1557*	*SCN2A*	1 case with seizure 1 case with ID and recurrent catatonia	4; 20	PCRS	Corticoids not very effective. Treated with lorazepam. IVIg not effective, lorazepam + ECT	[152, 153]
Hexanucleotide repeat expansion	*C9orf72*	2 cases with MDD.	65; 67	BFCRS	Antidepressant, Aripiprazole, Lorazepam, and ECT	[154–156]
14q11.2 duplication	*SUPTH16, CDH8*	1 case with schizophrenia and seizure	50	DSM–5	Clozapine	[38]
2q36.1 duplication	*SGPP2, FARSB, MOGAT1*	Concomitant with DS	/	DSM–5	ECT	[38]
16p11.2 duplication	*SLC7A5P1, SPN, QPRT, C16orf54, ZG16, KIF22, MAZ, PRRT2, MVP, CDIPT, SEZ6L2, ASPDH1, KCTD13, TMEM219, TAOK2, HIRIP3, INO80E, DOC2A, C16orf92, TLCD3B, CTD2515O10.6, PPP4C, TBX6, YPEL3, GDPD3, MAPK3*	1 case with neuro-syphilis, seizure and SSD	/	DSM–5	Lorazepam	[38]
14q11.2 duplication	*ZNF219, TMEM253, OR5AU1*, *HNRNPC, RPGRIP1, SUPT16H, CHD8*	1 case with seizure and SSD	/	DSM–5	Clozapine	[38]
Pathogenic variant c.2565del	*CHD8*	1 case of catatonia and regression following viral infection in ASD	13	/	Resistant to Lorazepam, ECT and immunomodulatory medications	[157]
16p13.13 duplication	*SNX29, CPPED1*	1 case with psychosis	/	DSM–5	Lorazepam	[38]
16p13.11p12.3 duplication	*NPIPA1, PDXDC1, NTAN1, RRN3, NPIPA5, MPV17L, BMERB1, MARF1, NDE1, MYH11, CEP20, ABCC1, ABCC6, NOMO3*	Chronic catatonia with skill regression, ASD and DI	21	/	/	[7]
17p13.3 duplication	*SLC43A2, SCARF1, PRPF8, RILP*	1 case with psychosis	/	DSM–5	ECT	[38]
Klinefelter extra X chromosome	Multiple	1 case with BP and MC	42	/	ECT	[73]
Missense mutation	*KCNT1*	5 cases	/	/	/	[158]
Nonsense mutation	*HARS*	1 case with psychosis	/	/	/	[159]
Missense mutation	*MMACHC*	2 cases, 1 with psychosis	15 ; 14	/	Betaine, hydroxycobalamine	[80, 81]
G6PD deficiency	*G6PD*	2 cases, 1 with mood disorder and recurrent catatonia, 1 with MC	35; 19	BFCRS	Lithium; ECT	[77]
Wilson disease	*ATB7B*	5 cases with MC	22 ; 12 ; 19 ; 19 ; 18	MRS ; BFCRS	Penicillamine Zinc BZD Lorazepam failed, treated with ECT	[87–90]
MTHFR deficiency	*MTHFR*	1 case with psychosis 1 case with MDD	34 ; 18	BFCRS	1 case treated with betaine 1 case with ECT and AD	[83, 84]
Acute porphyria	*HMBS*	2 cases with psychosis	46 ; 14	/	Treated with ECT	[91, 92]
Cerebrotendinous Xanthomatosis	*CYP27A1*	1 case with seizure	20	/	Chenodeoxycholic acid + HMG-CoA reductase inhibitor + valproate	[95]
Niemann-Pick Disease Type C heterozygous mutations	*NPC1*	1 case with recurrent catatonia and VSGP	23	BFCRS	Lorazepam paradoxal response Treated with ECT	[97]
GM2 gangliosidosis (Tay-Sachs disease)	*HEXA*	2 cases: 1 with SSD, 1 with ID	17 ; 14	/	Lorazepam	[99, 100]
Nonsense variant (SHINE syndrome)	*DLG4*	1 case early onset SSD	13	/	Clonazepam and clozapine	[160]
Variant of unknwon significance	*KIDINS220*	1 case chronic catatonia in ASD and DI	18	/	Clonazepam	[7]
Variant of unknwon significance	*SORCS1*	1 chronic catatonia with ASD DI and seizure	13	/	BZD and ECT	[7]
Variant of unknwon significance	*STAG1*	/	/	/	/	[7]
	*IQGAP3*					
	*STARD9*					
	*CSMD1*					
	*NAA15*					
2p16.3 deletion	*NRXN1*	Chronic catatonia with ASD DI	16	/	BZD	[7]
Kallmann syndrome	*PROK2*	Chronic catatonia in ASD DI seizure and regression	12	/	Lorazepam ECT	[7]
18p11.32p11.21	Multiple + pathogenic variant in TNFRSF13B	Chronic catatonia and ASD and DT1, hashimoto thyroidisme	17	BFCRS	Lorazepam ECT Then IVIg, mycocophenolic acide and hydroxychloroquine	[7]
Xp22.31 deletion	*PUPD, STS, VCX, PNPLA4*	Chronic catatonia regression and ASD	17	/	Lorazepam and ECT	[7]
Cohen Gibson syndrome	*EED*	Catatonia with overgrowth, ID, seizure	49	/	/	[161]
Pathogenic variant	*KCNQ1*	Catatonia and psychosis	15	/	Lorazepam	[162]
Duplication	*ADCY8*	Catatonia and ASD and psychosis	16	/	Lorazepam and ECT	[162]
3p26.3-p26.2 intragenic deletion and 2q21.1 duplication	*CNTN4 ; GPR39, LYPD1*	Catatonia and mood disorder	15	/	Lorazepam and ECT	[162]
mtDNA MELAS	MT-TL1	Acute catatonia post “stroke like episode”; acute catatonia and MDD	41; 17	DSM-IV	Lorazepam and haloperidol; escitalopram and methylphenidate	[107, 108]

*Note*: With ‘/’= not available or not relevant.

Abbreviations: ABQ, attenuated behavior questionnaire; ADHD, attention deficit hyperactive disorder; ASD, autism spectrum disorder; BFCRS, Bush Francis catatonia rating scale; BP, bipolar disorder; BZD, benzodiazepines; DS, Down syndrome; ECT, electroconvulsive therapy; ID, intellectual disability; IVIg, intravenous immunoglobulins; MC, malignant catatonia; MDD, major depressive disorder; MRS, modified Rogers scale; OCD, obsessional compulsive disorder; PCRS, pediatric catatonia rating scale; SSD, schizophrenia spectrum disorder; tDCS, transcranial direct current stimulation; VSGP, vertical supranuclear gaze palsy.

**Figure 2.**
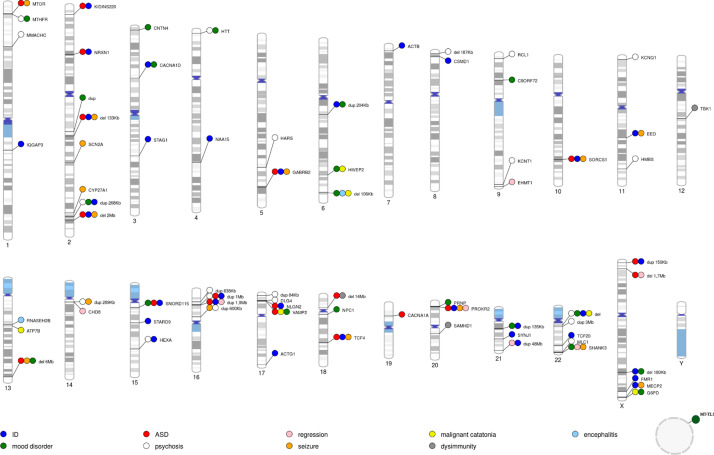
Location of genetic variants and small deletions and duplications reported in catatonia. The main phenotypes associated with catatonia are shown in color. The name of the gene or the size of the deletion or duplication is indicated next to it. ID = intellectual disability; ASD= autism spectrum disorder. The circle corresponds to mitochondrial DNA.

**Figure 3.**
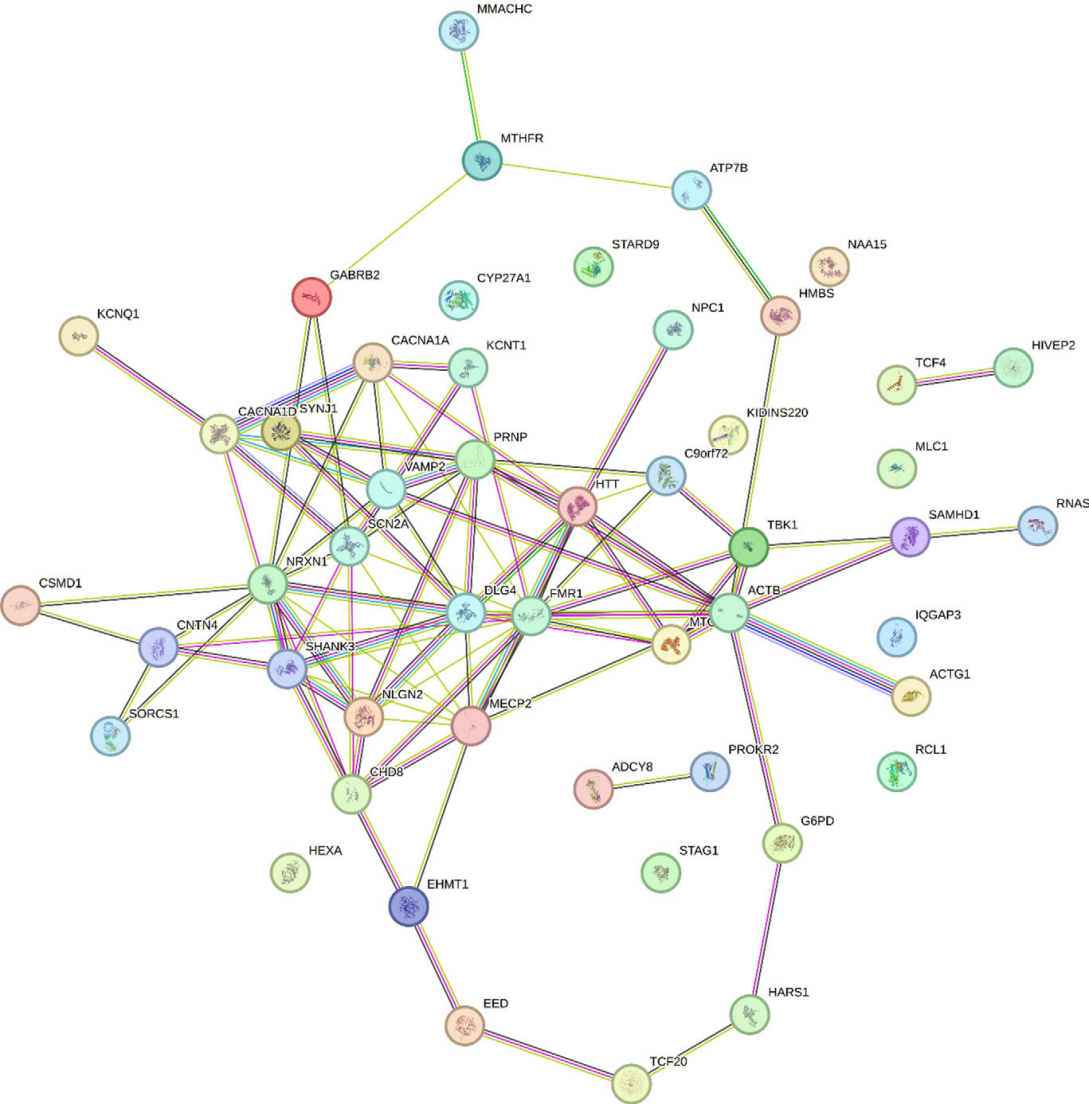
Gene network mapping for the 50 variants reported in catatonia.

**Figure 4.**
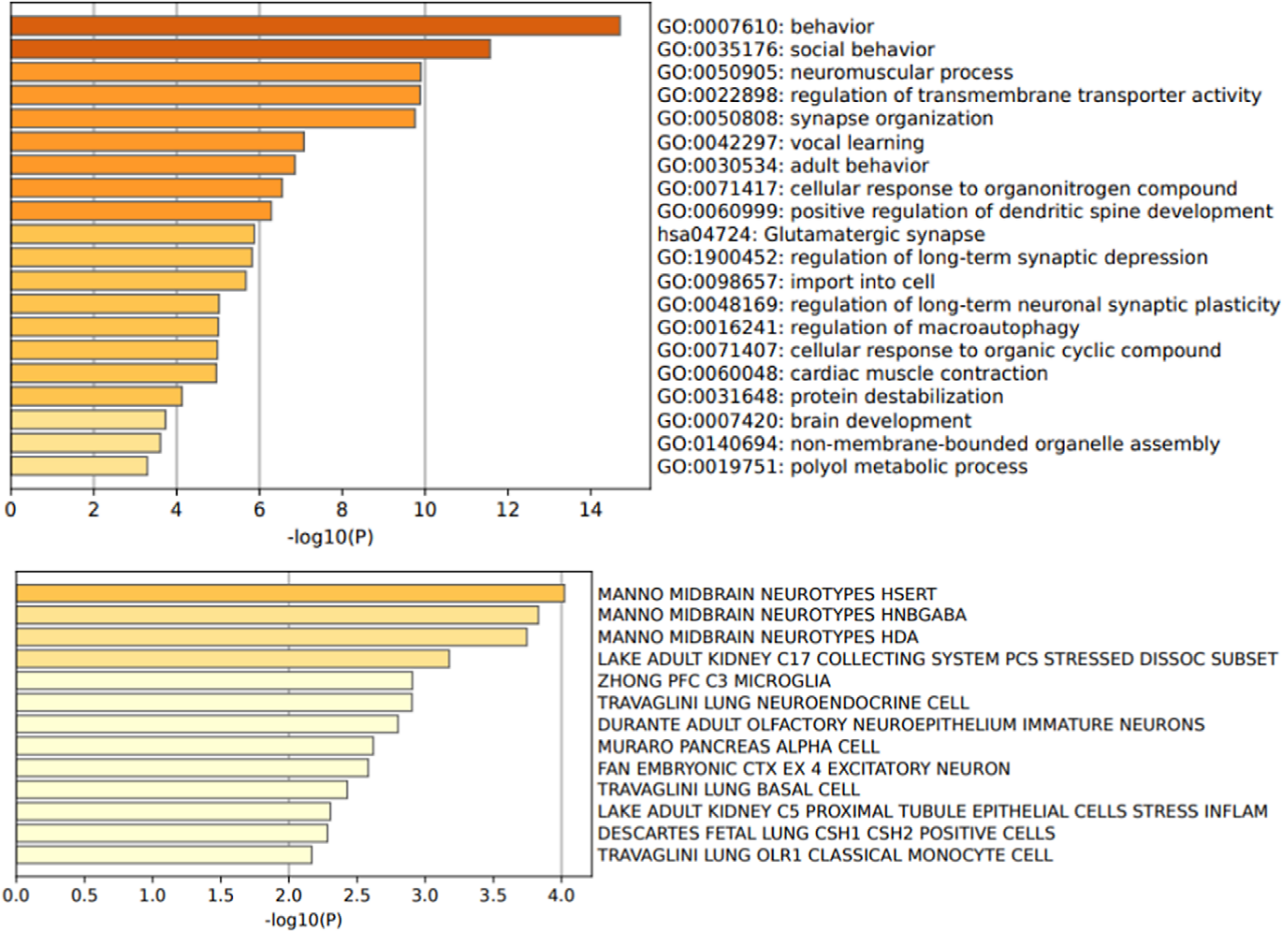
Gene ontology biological processes and cell type signatures of the 50 variants reported in catatonia. *p*-values are corrected for multiple testing and the colors represent the *p*-value magnitude, with darker shades indicating smaller *p*-values.

We used the PhenoGram software [27] (http://visualization.ritchielab.org/phenograms/plot) to represent graphically the genetic abnormalities linked to catatonia (Figure 2). We used the String software (http://string-db.org) for a map of interaction (Figure 3) and the Metascape software [28] (https://metascape.org) to identify the biological processes and the cell type enrichment (Figure 4).

## Results

Ninety-four articles met the criteria for our systematic review (Figure 1). This corresponds to 374 cases of catatonic syndrome and 78 different genetic abnormalities, as described in Table 1 and Figure 2. Below, we extensively describe the conditions where multiple cases of catatonia have been reported

### Down syndrome: trisomy 21

Patients with trisomy 21, or Down’s syndrome, which has an incidence of 1 in 800 births [29], are at risk of developing a catatonic syndrome. The risk of having a catatonic episode, which is often chronic, occurs during adolescence and is generally accompanied by behavioral regression [30, 31]. This behavioral regression, known as Down syndrome regression disorder, has been increasingly described in the literature in recent years, and a recent international experts consensus has established diagnostic criteria, including a criterion for catatonia [32]. Some studies highlight the effectiveness of immunomodulatory treatments, e.g. intravenous immunoglobulin (IVIg) infusions, which are effective in 85% of cases [33, 34]. This robust IVIg response rate is intricately linked to the well-documented autoimmune context inherent to Down syndrome [35]. Patients with Down syndrome regression disorder exhibit a heightened prevalence of autoimmune conditions compared to those with Down syndrome without regression, as evidenced by elevated inflammatory markers in their bloodstream [36]. A recent study has detected 365 auto-antibodies in the plasma of individuals with Down syndrome, some targeting the central nervous and immune system [35]. This increased vulnerability to catatonia in these patients reinforces the hypothesis of brain inflammation as a potential causative factor in catatonia [24, 25].

### Phelan-McDermid syndrome: SHANK3 gene

Phelan-McDermid syndrome is a rare condition characterized by deletion or mutation within the *SHANK3* gene in chromosome region 22q13.33. The prevalence of this syndrome is currently unknown. However, it is estimated that approximately 0.5% to 1% of ASD cases with intellectual disability are caused by PMS [16]. The prevalence of catatonia within this syndrome reaches 53% [37]. Fifty-one cases of Phelan-McDermid syndrome with catatonia have been reported in the literature see Table 1. Haploinsufficiency of *SHANK3* appears to be a risk factor for catatonic syndrome [38]. *SHANK3* encodes a scaffolding protein of the postsynaptic density of glutamatergic excitatory synapses. Deficiency of this protein causes hypofunction of NMDA receptors (NMDAR) [39]. The Phelan-McDermid syndrome with catatonia model further supports the overarching hypothesis of glutamatergic system hypofunctionality in catatonia, a concept advanced by several authors, especially concerning the intricate balance between GABA and glutamate neurotransmitters [23]. Furthermore, it is essential to note that all episodes of catatonia noted within Phelan-McDermid syndrome exhibit a chronic course (>12 weeks) and limited response to conventional therapeutic approaches. In this syndrome, benzodiazepines such as lorazepam can increase impulsivity, psychomotor excitation, confusion, and insomnia, limiting the use of this treatment [16]. Several alternative treatments have been tried in Phelan-McDermid syndrome with catatonia, such as lithium [40] and transcranial direct current stimulation (tDCS) in 4 patients with good efficacy and safety [41]. tDCS is postulated to enhance glutamatergic synaptic function through the induction of sustained long-term potentiation, thereby fostering metaplasticity that can persist for weeks [42].

### Di George syndrome: 22q11.2 deletion

Di George syndrome, also known as 22q11.2 deletion syndrome, occurs in approximately 1 in 4,000 births, and is characterized by a deletion of variable size in the 22q11.2 region [43]. A total of 21 cases of 22q11.2 deletion syndrome is reported in the literature (Table 1) is most likely underestimated, given the well-known prevalence of psychotic and mood disorders among 22q11 deletion syndrome [44], as well as various motor abnormalities [45]. A less expected case of catatonia has been described in a 22q11.2 duplication [38]. The management of catatonic syndrome in these patients appears to be challenging. In Butcher and colleagues’ case series, few patients were treated with the specific treatment for catatonic syndrome, i.e. lorazepam and ECT, and the efficacy was variable. Two were unsuccessfully treated with IVIg for suspected encephalitis, and four patients had a poor outcome, including malignant catatonia [21]. The other case report shows a good response to ECT but highlights the difficulty of diagnosing catatonia with BFCRS in these patients with 22q11.2 deletion syndrome [46]. The 22q11.2 region involves about 90 genes, including 46 protein-coding genes, pseudogenes, non-coding RNAs, and microRNAs [47], in particular, the *PRODH* gene, which encodes a mitochondrial enzyme involved in balancing GABA/glutamate transmission [21, 48]. A case of catatonia associated with a missense mutation in the *PRODH* gene has been reported in the literature [8] (Table 1). This gene may be essential in developing catatonic symptoms in patients with 22q11.2 deletion syndrome. In addition, immune dysregulation has been highlighted in this syndrome, which appears to be associated with neuropsychiatric manifestations with elevated levels of pro-inflammatory cytokines, complement activity, increased neutrophils, and blood-brain barrier dysfunction [25]. This underlying neuroinflammation presents an additional avenue of risk for catatonia development, potentially synergizing with the influence of the *PRODH* gene.

### Kleefstra syndrome: EHMT1 gene

Kleefstra syndrome emerges from the haploinsufficiency of *EHMT1* due to either a deletion at 9q34.3 or pathogenic variants of *EHMT1* [49]. The prevalence is unknown [49]. Six cases of catatonia in Kleefstra syndrome have been described in the literature (Table 1) in the context of regression of abilities. Little is known about the therapeutic approach for catatonia in these patients. *EHMT1* orchestrates histone methylation and facilitates gene silencing. Alongside *EHMT2*, it plays a pivotal role in synaptic scaling [50]. A study involving excitatory cortical neurons derived from induced pluripotent stem cells obtained from Kleefstra syndrome patients exhibited *EHMT1* deficiency-triggered NMDAR hyperactivity [51]. Furthermore, insights from a Kleefstra syndrome mouse model illuminated elevated expression of specific inflammatory genes, including IL-1b, alongside an increase in activated microglial cells within the brain, thereby underscoring the presence of cerebral inflammation in these patients [52]. The predisposition to catatonia within Kleefstra syndrome might be linked to the perturbation of the E/I balance within a neuroinflammatory cerebral milieu.

### Prader-Willi syndrome: 15q11-q13 region

Catatonia has been reported in Prader-Willi syndrome, associated with lack of expression of paternally inherited imprinted genes in the chromosome 15q11-q13 region generally caused by a paternal deletion or maternal disomy in with both chromosomes 15 being inherited from the mother [53]. Prader-Willi syndrome occurs in approximately 1 in 15,000 individuals. Neuropsychiatric features are common in this condition, with anxiety and compulsive behaviors being the most prevalent [54]. The use of lorazepam can be challenging in these patients with extreme obesity and obstructive sleep apnea syndrome with a higher risk of dyspnea [53]. ECT treatment seems to be somewhat effective [55, 56]. The gene region implicated in Prader-Willi syndrome also includes the brain-specific, non-coding, Small Nucleolar Ribonucleic acid C/D box 115-1(SNORD 115). SNORD 115 dysfunction, reported in 3 case reports [57], is hypothesized to contribute to catatonia across various neuropsychiatric disorders, including autism, schizophrenia, bipolar disorder, and major depressive disorder, as well as genetic and immune-related conditions through the regulation of five downstream genes: CRHR1, PBRM1, TAF1, DPM2, RALGPS1 and the alternative splicing of serotonin 2C receptor [57]. TAF1 and DPM2 provide potential clues about parkinsonism and increased creatine phosphokinase in neuroleptic malignant syndrome, while abnormalities in RALGPS1 suggest links to both anti-NMDAR antibody encephalitis and the SHANK3 gene, which is known to predispose to catatonia.

### Huntington’s disease: HTT gene

Huntington’s disease, caused by an expanded CAG trinucleotide repeat in the *HTT* gene encoding a non-functional huntingtin protein [58], is often associated with psychiatric features in addition to motor dysfunction. It occurs approximately in one in 7,300 individuals [58]. Catatonia in Huntington’s disease is described in five cases in the literature in the context of concurrent schizophrenia and mood disorders with good response to ECT. ECT should be widely offered to patients with catatonia in Huntington’s disease. However, the diagnosis is difficult to make in patients whose psychiatric manifestations precede their motor symptoms by several years [59].

### X-linked syndrome

#### Fragile X syndrome: FMR1 gene

Fragile X syndrome is related to a number of CGG trinucleotide repeats >200 in the FMR1 gene’s promoter region. The prevalence of the full Fragile X syndrome mutation in the general population is estimated to be approximately 1 in 5,000 males and between 1 in 4,000 and 1 in 8,000 females [60]. Expansions between 55 and 200 repeats are defined as a premutation, and its relationship with neuropsychiatric symptoms is discussed [61, 62]. A case series described eight patients with Fragile X syndrome with “catatonia-like behaviour” scored by the attenuated behaviour questionnaire (ABQ) [63]. Unfortunately, treatment was not described in this study. In addition, the ABQ scale used, which was developed to describe patients with ASD and catatonic symptoms, seems to overestimate the prevalence of catatonia and, to be less specific, with correlations with depression and repetitive/restrictive behavior. Moreover, a case of Fragile X syndrome and a neuro-lupus with catatonia is reported with a good response to ECT [64]. A male with a premutation has been described alongside ASD and ADHD associated with catatonia and a good response to ECT [65]. ECT appears to be well tolerated and effective in Fragile X syndrome or premutation careers – the FMR1 expansion results in reduced levels of Fragile X mental retardation protein (FMRP). The absence of FMRP leads to the downregulation of GABA-A and B receptors and the upregulation of glutamate receptors. This disruption in the balance of GABA/glutamate levels could likely predispose these patients to catatonia [65].

#### Rett syndrome: MECP2 gene

Rett syndrome results from a methyl CpG binding protein 2 (MECP2) gene mutation. This disorder predominantly impacts girls, accounting for 95 to 97% of cases and has a prevalence of fewer than 1 in 200,000 individuals [66]. Its hallmark features encompass developmental regression coupled with movement disorders, notably hand stereotypies [67]. Within Rett syndrome, an array of additional movement disorders has been documented, including a rigid akinetic syndrome prevalent in older patients. One case of catatonia in a primary school-aged girl with Rett syndrome was associated with new-onset incontinence in addition to posturing, waxy flexibility, rigidity, staring and grimacing and was resolved with 8mg of lorazepam [2]. Catatonia has been described in two 17-year-old males with MECP2 deficiency [68, 69]. In males carrying *MECP2* variants, intellectual deficiency is associated with parkinsonism features [68]. One presented with a catatonic episode resolved with lorazepam, alongside a vertical supranuclear gaze palsy [68]; the other had recurrent lorazepam-resistant catatonia and was successfully treated with tDCS [69]. A noteworthy conjecture arises that several individuals with Rett syndrome and presenting parkinsonism features might be misdiagnosed and potentially could be suffering from catatonia. Using the ABQ scale, 32 patients diagnosed with Rett syndrome were evaluated [70]. Of this group, 20 exhibited catatonia-like behaviors. Rett syndrome is primarily a synaptic disorder, and MECP2 deficiency impacts excitatory and inhibitory synapses, leading to an elevated E/I ratio in animal model [71].

#### Klinefelter syndrome: extra X chromosome

Klinefelter’s syndrome arises from the presence of one or more additional X chromosomes (i.e. 47XXY). Its incidence is approximately 1 in 750 births [72]. A case of catatonia is described in a 42-year-old patient with Klinefelter’s syndrome and bipolar disorder [73]. The presentation was rather severe, with malignant catatonia coupled with respiratory failure and effectively treated with ECT. Association between the extra X chromosome and psychiatric disorders has long been known with, in particular, an increased risk of psychotic disorders [74]. However, very few cases of Klinefelter syndrome with catatonia have been reported, probably due to the underdiagnosis of both Klinefelter syndrome and catatonia.

### Inborn errors of metabolism

Inborn errors of metabolism are linked to genetic mutations, resulting in deficiencies in metabolic enzymes and leading to the accumulation or reduced excretion of protein, carbohydrates, and lipids. These diseases are most often diagnosed in early childhood, although milder forms occurring in adolescence and adulthood may be marked by psychiatric manifestations [75]. Inborn errors of metabolism are individually rare, however, more than 1,000 types have been identified, with a combined prevalence of approximately 1 in 800 to 1 in 1,000 individuals [75]. Lahutte and colleagues have compiled a list of inborn errors of metabolism likely associated with catatonia [76]. We present inborn errors of metabolism and catatonia cases documented in the literature (Table 1). Two cases of G6PD deficiency were associated with catatonia [77] and, in particular, one with malignant catatonia requiring ECT. G6PD deficiency has been associated with psychiatric disorders, notably psychosis and mood disorders [78]. It is relatively common, with an estimated global prevalence of over 500 million individuals [79]. Among treatable diseases, two cases of cobalamin C deficiency leading to hyperhomocysteinemia presented with catatonia in two girls of 10 and 15 years old [80, 81]. In both cases, catatonia regressed with the specific treatment of hydroxocobalamin, betaine and carnitine. Cobalamin C deficiency occurs in approximately 1 in 100,000 live births [82]. In addition, 2 cases of MTHFR deficiency also led to hyperhomocysteinemia in two girls, 34 and 18 years old. One was treated with betaine, yielding limited effectiveness, while the other underwent ECT [83, 84]. MTHFR deficiency is the most frequent folate metabolic disorder although the incidence is very rare( <1/400,000) [85]. Regarding Wilson’s disease, with an estimated incidence of ~1 per 7,000 individuals [86], five cases of catatonia have been reported, including one marked by malignant catatonia [87–90]. These cases raised diagnostic challenges, and in four cases, identifying the Kayser–Fleischer ring facilitated accurate diagnosis and the proposal of targeted treatments. Indeed, cases were effectively treated with the specific therapy based on penicillamine and zinc in addition to a benzodiazepine, with one case necessitating ECT. In addition, two cases of acute porphyria, with catatonia alongside psychosis exhibited significant improvements with ECT [91, 92]. Acute porphyria has an estimated incidence of 10 per million [93]. A case of cerebrotendinous xanthomatosis, incidence 1 in 50,000 [94], and catatonia was effectively treated with the specific treatment of chenodeoxycholic acid and HMG-CoA reductase inhibitor coupled with valproate for seizure control [95]. A case of Niemann-Pick type C disease, incidence 0.82/100,000 [96], is reported in a 23-year-old boy who exhibited recurrent catatonia alongside neurological manifestations like vertical supranuclear gaze palsy [97]. Finally, two cases of GM2 gangliosidosis, also known as Tay–Sachs disease, incidence of one in 320,000 births [98], were reported, and catatonia in these cases was alleviated with lorazepam [99, 100].

### Interferonopathies

Catatonia has been described in two patients with Aicardi–Goutières syndrome [101, 102]. Aicardi-Goutières syndrome is an autosomal recessive encephalopathy within the type 1 interferonopathies characterized by increased type 1 interferon (IFN) signaling[103] with an incidence less than 0.7600/100,000 live births [104]. Both cases presented with neurodevelopmental delay associated with bilateral basal ganglia calcifications on CT scan. One had typical skin lesions. In one case, the catatonic syndrome was successfully treated with immunoadsorption (22 treatments over 8 weeks) [101]. Another case of catatonia was described in a woman with post-infectious psychosis and a TBK1 variant [105]. TBK1 upregulates type 1 IFN transcription genes. Type 1 IFN is essential as an antiviral cytokine and regulates innate and adaptive immune responses. Dysregulation of IFN signaling could play a key role in triggering the cerebral inflammation that leads to catatonia [25].

### Mitochondrial DNA mutation

Catatonia has been documented in two cases involving mitochondrial DNA mutation, the MELAS (mitochondrial encephalopathy with lactic acidosis and stroke-like episodes) syndrome, prevalence of 16 to 18/100,000 [106]. A 41-year-old woman presented with catatonia following stroke-like episodes (SLE) [107], a hallmark symptom of MELAS driven by ictal activity leading to a range of neurological and psychiatric manifestations. The catatonic episode was successfully resolved with benzodiazepines. In another case, a patient with MELAS exhibited acute catatonia in the context of a major depressive disorder, which responded well to antidepressant treatment [108]. Psychiatric symptoms are prevalent in mitochondrial disorders ranging from 6 to 28%, including anxiety, mood, and psychosis disorders [109], and are likely to be related to a dysfunction of the brain’s respiratory chain. Antipsychotics must be cautiously considered, both typical and atypical, as they have been shown to inhibit complex I of the respiratory chain, potentially exacerbating the symptoms [109].

### Clinical and therapeutic overview

Of 353 cases with a complete clinical description, 241 cases (68%) had a chronic or recurrent presentation, and 14 cases (4%) presented with malignant catatonia. Of 311 cases with documented treatment data, 261 cases (84%) showed a poor response to lorazepam, and 135 cases (43%) were treated with ECT, in contrast to the typical 70% good response rate to lorazepam in catatonia [1]. Only three were reported to be treated with clozapine.

### Enrichment analyses

This systematic literature review identified 50 genetic variants associated with catatonia alongside 23 small-scale duplications and deletions. In our analysis, we have excluded genes encompassed by deletions and duplications, as it is challenging to determine which subset of genes plays a significant role in catatonia. The 50 genetic variants exhibit high interconnectivity, suggesting a potential convergence of shared biological processes, as illustrated in Figure 3. The protein-protein interaction revealed pathway enrichment in postsynaptic membrane organization (*p* = 6.31×10^−13^), positive regulation of excitatory postsynaptic potential (*p* = 10–12), and receptor clustering (*p* = 5×10^−12^). The variants are enriched in genes involved in behavior (*p* = 2 × 10^−15^), social behavior (*p* = 2 × 10^−12^), regulation of transmembrane transporter activity (*p* = 3.63 × 10^−11^), synapse organization (*p* = 1.2 × 10^−10^) and neuromuscular process (*p* = 1.1 × 10^−10^) (Figure 4). These genes are mainly expressed in GABAergic (*p* = 1.5 × 10^−4^), serotoninergic (*p* = 1 × 10^−4^), dopaminergic (*p* = 2 × 10^−4^), and excitatory (*p* = 1.6 × 10^−3^) neurons as well as in microglial cells (*p* = 5 × 10^−3^) (Figure 4).

## Discussion

In this review, we reported all the genetic anomalies associated with a catatonic syndrome described in the literature to date. This analysis included 94 articles with 374 cases of catatonia and 78 distinct genetic anomalies.

Firstly, regarding clinical presentation, this review highlights that catatonic episodes associated with genetic abnormalities exhibit a chronic course in 241 cases (68%). Additionally, typical clinical signs of catatonia may be absent, with behavioral regression often being a predominant feature. A recent review article proposes assessing a personalized score at baseline based on the BFCRS and the pediatric catatonia rating scale (PCRS) [163] to facilitate diagnosis and assessment of response to treatment [2]. In addition, some cases of catatonia with genetic abnormalities have been described in neurodegenerative disorders such as *C9orf72*, associated with frontotemporal dementia, and in Huntington’s disease, reinforcing the importance of looking for genetic abnormalities also in neurodegenerative disorders in line with the known associated vulnerability between neurodevelopmental and neurodegenerative disorders [164].

Concerning the therapeutic aspect, in the majority of cases (261 cases; 84%), lorazepam proved inadequate in alleviating catatonia, exhibiting reduced efficacy or poor tolerance. Electroconvulsive therapy was frequently used (135 cases; 43%) and was mostly effective but limited by its restrictive access [15, 165]. Thus, although ECT should be widely offered [166], alternative treatments must be discussed. Although only three cases of catatonia were treated with clozapine with significant improvement, clozapine appears to be well tolerated and effective in patients with neurodevelopmental disorders [167] and should therefore be part of the personalized care strategies considered. The efficacy of clozapine may be closely linked to the imbalance in the E/I ratio and, in particular, to the involvement of gabaergic interneurons [168, 169]. Indeed, clozapine may facilitate the binding of GABA to the GABA_B_ receptor [170]. tDCS, an easy-to-apply noninvasive brain stimulation technique, was effective and safe in 16 cases of catatonic patients, including four patients with Phelan-McDermid syndrome [41, 171] and two patients with Down Syndrome Regression Disorder [172]. In addition, in catatonia cases related to neuroinflammation, anti-inflammatory interventions appear promising [34, 173]. Anti-inflammatory treatment needs to be offered more widely, even in the absence of autoantibodies identified by lumbar puncture or blood test, in syndromes with known inflammation such as Down Syndrome Regression Disorder [33]. Along the same lines, specific treatment in case of treatable inborn errors of metabolism, such as Cobalamin C disorder or Wilson disease, can treat catatonia. Knowledge of genetic abnormalities can help organize timely personalized care [41, 140]. Likewise, it is legitimate to systematically assess catatonic symptoms in genetic diseases, notably in Down syndrome and Phelan–McDermid syndrome, where its prevalence is extremely high [32, 37].

In light of this literature review, genetic testing for catatonia should be considered more broadly in patients with the following criteria: the presence of a neurodevelopmental background [7], a chronic episode lasting more than 12 weeks, recurrent episodes, early onset of catatonia, resistance or paradoxical reaction to first-line treatment of catatonia, association with neurological signs including seizures, and the existence of malignant catatonia. Whole-exome or whole-genome sequencing should be considered the first-tier diagnostic framework, considering the high proportion of SNVs.

Although the risk of developing catatonia is high in certain genetic conditions, the onset of a catatonic episode may depend on environmental factors, such as exposure to antipsychotics [17] or the sudden discontinuation of clozapine treatment [174]. Well-conducted studies are needed to identify environmental risk factors in these genetically predisposed patients to develop effective prevention strategies.

Finally, a deeper understanding of the genetic factors involved in catatonia can shed light on its pathophysiology. The protein–protein interaction, enrichment in biological processes, and the cell type signatures in the 50 genetic variants associated with catatonia reinforce the hypothesis of a dysfunction at the synaptic level, particularly in GABAergic neurons. It supports the likely central role of GABAergic interneurons in catatonia [174, 175] in the hypothesis of E/I imbalance [23]. Glutamate modulates the activity of the GABAergic interneurons through their NMDA receptors that regulate the synchronization of superficial pyramidal cells, thus ensuring a variety of cognitive processes [176]. A disruption of GABAergic interneurons has been highlighted in several psychiatric disorders, including ASD and schizophrenia [176, 177], both of which are associated with catatonia. This disruption may underlie the dysconnectivity between the medial prefrontal, orbitofrontal, and motor areas observed in fMRI studies of catatonia [178] and could represent a shared pathophysiological mechanism linking these disorders to catatonia. Furthermore, as interneuron-dependent prefrontal cortical maturation occurs during adolescence [179], their disruption may also explain the specific timing of catatonia onset in syndromes such as DSRD. Adolescence thus emerges as a vulnerable timeframe for catatonia. Quantitative EEG analyses could be used to provide further support for the hypothesis of disruption of GABAergic interneurons in catatonia [180]. Such anomalies in GABAergic function could be due to various pathological processes, contributing to the diverse clinical presentations observed in catatonia. One potential mechanism is neuro-inflammation, a phenomenon observed in several genetic diseases, notably Down’s syndrome and 22q11DS, and also reported in catatonia [24, 25]. Autoantibodies targeting GABAergic interneurons could lead to catatonia, as it has also been observed for autoantibodies targeting NMDA receptors [181]. The microglial cell signature of genetic variants associated with catatonia further underscores the involvement of neuroinflammation. Based on our cell-enrichment analysis, serotonergic and dopaminergic neurons also appear to be involved. Serotonin receptors have previously been associated with catatonia, notably via SNORD 115 mRNA, which regulates alternative splicing of the serotonin 2C receptor [57]. Furthermore, catatonia is frequently associated with both serotonin syndrome and neuroleptic malignant syndrome, each respectively associated with an excess of serotonin and dopamine blockage [182].

Several methodological issues call for caution when interpreting this work. First, catatonia is not always described using the same scale, and sometimes no scale has been used. It is rather difficult to ascertain the diagnosis and severity of the syndrome. Second, we probably missed genetic abnormalities as the vast majority of genetic cases with catatonia have not been published, and there is still limited access to genetic testing in psychiatric disorders. Another form of bias stems from the limited conclusiveness of cases due to our current understanding of genetics. This understanding is influenced by variants found in other psychiatric conditions, resulting in the expectation that biological processes and cell signatures will be concentrated in usual brain pathways. In addition, given the absence of a control group, we cannot ascertain whether these genetic variations are truly enriched in catatonia specifically or if their presence merely reflects broader neurodevelopmental processes associated with catatonia. Nevertheless, these findings provide a valuable foundation for generating research hypotheses. To overcome this limitation, future studies should incorporate larger, well-controlled, and unbiased genetic analyses involving patients with catatonia.

## Conclusion

This review highlighted the neurodevelopmental burden of catatonia and the clinical relevance of genetic explorations in this syndrome while also discussing the underlying pathophysiology and its implications for treatment. Our systematic description of rare disorders associated with catatonia, providing clear links between genotype and phenotype, strengthens the importance of GABAergic interneuron dysfunction. This work further points out the importance of large-scale whole-exome or whole-genome sequencing studies to identify new genetic variants and pathways associated with catatonia. Improving our knowledge of catatonia’s genetic background may allow for the development of more targeted diagnostic approaches and personalized treatment strategies.

## Data Availability

All the data are available.
